# Identifying transdiagnostic biological subtypes across schizophrenia, bipolar disorder, and major depressive disorder based on lipidomics profiles

**DOI:** 10.3389/fcell.2022.969575

**Published:** 2022-09-05

**Authors:** Shiwan Tao, Yamin Zhang, Qiang Wang, Chunxia Qiao, Wei Deng, Sugai Liang, Jinxue Wei, Wei Wei, Hua Yu, Xiaojing Li, Mingli Li, Wanjun Guo, Xiaohong Ma, Liansheng Zhao, Tao Li

**Affiliations:** ^1^ Mental Health Center and Psychiatric Laboratory, West China Hospital of Sichuan University, Chengdu, Sichuan, China; ^2^ Department of Neurobiology, Affiliated Mental Health Center & Hangzhou Seventh People’s Hospital, Zhejiang University School of Medicine, Hangzhou, Zhejiang, China; ^3^ NHC and CAMS Key Laboratory of Medical Neurobiology, MOE Frontier Science Center for Brain Science and Brain-Machine Integration, School of Brain Science and Brain Medicine, Zhejiang University, Hangzhou, Zhejiang, China; ^4^ Guangdong-Hong Kong-Macao Greater Bay Area Center for Brain Science and Brain-Inspired Intelligence, Guangzhou, China

**Keywords:** schizophrenia, bipolar disorder, major depressive disorder, lipidomics profile, brain whiter matter

## Abstract

Emerging evidence has demonstrated overlapping biological abnormalities underlying schizophrenia (SCZ), bipolar disorder (BP), and major depressive disorder (MDD); these overlapping abnormalities help explain the high heterogeneity and the similarity of patients within and among diagnostic categories. This study aimed to identify transdiagnostic subtypes of these psychiatric disorders based on lipidomics abnormalities. We performed discriminant analysis to identify lipids that classified patients (N = 349, 112 with SCZ, 132 with BP, and 105 with MDD) and healthy controls (N = 198). Ten lipids that mainly regulate energy metabolism, inflammation, oxidative stress, and fatty acylation of proteins were identified. We found two subtypes (named Cluster 1 and Cluster 2 subtypes) across patients with SCZ, BP, and MDD by consensus clustering analysis based on the above 10 lipids. The distribution of clinical diagnosis, functional impairment measured by Global Assessment of Functioning (GAF) scales, and brain white matter abnormalities measured by fractional anisotropy (FA) and radial diffusivity (RD) differed in the two subtypes. Patients within the Cluster 2 subtype were mainly SCZ and BP patients and featured significantly elevated RD along the genu of corpus callosum (GCC) region and lower GAF scores than patients within the Cluster 1 subtype. The SCZ and BP patients within the Cluster 2 subtype shared similar biological patterns; that is, these patients had comparable brain white matter abnormalities and functional impairment, which is consistent with previous studies. Our findings indicate that peripheral lipid abnormalities might help identify homogeneous transdiagnostic subtypes across psychiatric disorders.

## 1 Introduction

Schizophrenia (SCZ), bipolar disorder (BP), and major depressive disorder (MDD) are three common psychiatric disorders with a heavy disease burden (Vigo et al., 2016). In clinical practice, it is an issue that the boundaries among the various diagnoses are not clearly distinct from each other. Patients with different diagnoses usually present baffling similarities to each other, such as the “with psychotic features” item of BP and MDD diagnoses and the emotional dysfunctions in SCZ. This is partly because rather than diagnostic objective criteria or biological markers, the current psychiatric diagnosis nosology relies on descriptive information elicited from self-report history and clinical observation (Scadding, 1996; Craddock and Mynors-Wallis, 2014; Heckers and Kendler, 2020). This hampers the diagnostic accuracy of these psychiatric disorders.

Increasing evidence has demonstrated that there are overlapping biological characteristics across SCZ, BP, and MDD, such as genetic risk factors (Cross-Disorder Group of the Psychiatric Genomics, 2013; Cross-Disorder Group of the Psychiatric Genomics et al., 2013; Ruderfer et al., 2014; Cross-Disorder Group of the Psychiatric Genomics Consortium. Electronic address and Cross-Disorder Group of the Psychiatric Genomics, 2019; Andlauer et al., 2021), brain structure and functional abnormalities (Meda et al., 2014; Godwin et al., 2018; Kelly et al., 2018; Li et al., 2018; Favre et al., 2019; van Velzen et al., 2020) and cognitive impairment (Czobor et al., 2007; Reichenberg et al., 2009; Millan et al., 2012; Barch and Sheffield, 2014; Reilly and Sweeney, 2014; Tamminga et al., 2014). All of these findings imply that the diagnostic classes are not distinct entities, and the descriptive diagnosis nosology has fundamental flaws. Therefore, it is necessary to identify natural biological homogeneous subtypes across different psychiatric disorders.

Lipid metabolites are downstream biochemical end products that are more close to phenotypes than genomics and proteomics. As an essential part of systems biology, lipidomics could comprehensively illuminate the lipid metabolic profile of individuals and identify changes related to phenotype (Patti et al., 2012; Zhao et al., 2014). Plasma lipid alterations, therefore, are sensitive and specific to several observed risk factors for psychiatric disorders, including genetic variations, brain white matter (WM) structural abnormalities, and oxidative stress and inflammation. For example, the ABCD1 gene mutation caused very long-chain fatty acid accumulation in brain WM, which led to psychiatric symptoms (Kitchin et al., 1987; Kemp et al., 2016). Plasma lipids, such as triglyceride, were also reported to be associated with brain WM microstructural changes and axonal degeneration (Iriondo et al., 2021). Notably, derived from peripheral essential omega-6 and omega-3 polyunsaturated fatty acids, lipid-derived mediators serve as pro/anti-inflammatory mediators regulating brain inflammation (Laye et al., 2018). Brain tissues are susceptible to oxidative stress due to their high oxygen consumption and unsaturated fatty acid enrichment, which have been reported to be associated with SCZ, BP, and MDD (Salim, 2017; Cobley et al., 2018).

Lipidomics has recently been developed as a powerful tool to investigate the natural characteristics of SCZ, BP, and MDD. The peripheral lipidomics profile alterations of these psychiatric disorders have been pervasively characterized using ultrahigh-performance liquid chromatography-tandem mass spectrometry (UHPLC–MS/MS) technology and have served as promising biomarkers for early diagnosis and clinical outcome prediction (Oresic et al., 2011; Brunkhorst-Kanaan et al., 2019; Zhou et al., 2019; Bot et al., 2020; Hussain et al., 2020; Zhuo et al., 2020; Dickens et al., 2021). Therefore, taking advantage of lipidomics analysis may help identify biologically homogeneous subtypes across these psychiatric disorders. In this study, first, we investigated the peripheral lipidomics profile abnormalities between psychiatric patients (with SCZ, BP, and MDD) and healthy controls (HCs) by discriminant analysis, and identify the most contributory lipids for classification. Then, based on these identified lipids, we further investigated the potential subtypes across SCZ, BP, and MDD by consensus clustering analysis. To comprehensively profile the differences in these potential biological subtypes, we further described and compared the brain WM microstructure and clinical features of these subtypes.

## 2 Methods

### 2.1 Participants

All participants, who were right-handed Han Chinese and aged 16–55 years old, were interviewed by at least two trained psychiatrists using the Structured Clinical Interview of the Diagnostic and Statistical Manual of Mental Disorders, 4th Edition, Text Revision (DSM-IV-TR)—Patient Version (SCID-P). A total of 547 participants (112 patients with SCZ, 132 with BP, 105 with MDD, and 198 HCs) were recruited from West China Hospital of Sichuan University between 2014 and 2019. The inclusion criteria for patients were as follows: 1) fulfilment of one of the DSM-IV-TR criteria for SCZ, BP, or MDD; 2) Han Chinese; 3) right-handed; 4) education achievement of more than 6 years; and 5) scores on Wechsler’s intelligence test equal to or higher than 70. The exclusion criteria for patients were as follows: 1) comorbidity with other DSM-IV-TR axis I or axis II disorders (such as alcohol and substance abuse); 2) presence of organic brain diseases, neurological diseases or somatic diseases undergoing drug treatment (such as diabetes); 3) any history of head trauma; 4) any physical therapies, such as electroconvulsive therapy, undergone within the past 6 months before magnetic resonance imaging (MRI) scan; 5) any contraindication to perform MRI scan; 6) pregnant or breastfeeding; and 7) Wechsler’s intelligence test scores less than 70. In this study, all SCZ patients were first-episode and drug-naïve. There were 77 MDD patients and 63 BP patients who were drug-naïve, and 28 MDD and 69 BP patients who were not drug-naïve but had at least a two-week wash-out period.

HCs were enrolled via online and local advertisements. They were screened for any mental disorder by the SCID—Non-Patient Version (SCID-NP). The exclusion criteria for HCs were similar to those for patients. Moreover, HCs with first-degree relatives with DSM-IV-TR axis I or II disorders were excluded.

### 2.2 Ethical principles

This study abided by the guidelines of the Declaration of Helsinki and was approved by the Institutional Ethics Committee of West China Hospital, Sichuan University. After the study procedure had been fully explained, written informed consent was obtained from all participants and their guardians if participants were less than 18 years old.

### 2.3 Clinical assessment

We used the Global Assessment of Functioning (GAF) scale to evaluate functional impairment in all patients. The Positive and Negative Syndrome Scale (PANSS), the Young Mania Rating Scale (YMRS), the Hamilton Anxiety Scale (HAMA), and the Hamilton Depression Scale (HAMD) were used to assess symptom severity in patients as appropriate. Clinical features, including onset age, total duration of illness period (TDP), current duration of illness period (CDP), duration of untreated period (DUP), current episode state, BP I or II subtype for BP, and the number of episodes for MDD, were also documented.

### 2.4 Lipidomics data acquisition and preprocessing

Peripheral blood was collected in EDTA tubes from all participants on the same day they were enrolled in this study. Lipid extraction, UHPLC‒MS/MS analysis, and lipid qualitative and quantitative identification are described in the Supplementary Methods. A total of 7212 lipid features in the positive polarity model and 4,898 lipid features in the negative polarity model were obtained. The lipidomic data were preprocessed by the “statTarget” (version 1.22.0) (Luan et al., 2018) and “MetaboAnalystR” (version 3.0.3) (Pang et al., 2020) packages in R software (version 4.1.0). We performed preprocessing steps as follows: 1) drift signal correction using the quality control-based robust locally estimated scatterplot smoothing (LOESS) signal correction (QC-RLSC) algorithm (Dunn et al., 2011); 2) a quality assurance procedure to remove metabolic features with relative standard deviation (RSD) >20%, which was calculated for all QC samples (Dunn et al., 2011); 3) log2 transformation and ComBat batch effect correction (Johnson et al., 2007); and 4) interquartile range (IQR) data filtering. The quality control results of the lipidomics data are described and depicted in Supplementary Figure S1. After the preprocessing steps, a total of 1,164 lipids remained for discriminant analysis.

### 2.5 DTI data acquisition and preprocessing

Brain WM microstructural abnormalities were measured by fractional anisotropy (FA) and radial diffusivity (RD) using diffusion tensor imaging (DTI) scans. The FA indicates the underlying characteristics of white matter microstructure, such as the directionality of axonal fibres, diameter, and density (Basser, 1995; Basser and Pierpaoli, 1996). RD is considered an indicator of myelin sheath thickness, reflecting myelin damage (Song et al., 2002; Song et al., 2005). Altered FA or RD in some regions indicated the brain white matter microstructures abnormalities here. The DTI scan parameters are described in the Supplementary Methods. Raw images were processed by MRIcroN (http://www.mricro.com), DTIPrep, and FMRIB Software Library (FSL) (version 5.0.8). The imaging format was converted by MRIcroN, and then the imaging quality was checked by DTIPrep (translation <2 mm, rotation <0.5 mm). Individual images that met the quality control criteria were included for subsequent procedures (6 samples were excluded after checking the imaging quality). The preprocessing steps included motion and eddy current correction, gradient direction reorientation, and brain mask estimation to remove the nonbrain spaces. After calculating diffusion tensor metrics, normalization and linear/nonlinear registration were also performed to allow comparison across participants. Brain regions of interest (ROIs) were defined by the JHU-ICBM-DTI-81 WM labels atlas (*n* = 48). The z scores of the mean FA and RD in each ROI were calculated for further statistical analysis.

### 2.6 Statistical analysis

#### 2.6.1 Demographic characteristics

Demographic characteristics including age, sex, educational attainment (years), and body mass index (BMI) of different groups (psychiatric patients and HCs) were compared using the independent test or chi-square test. BMI was calculated as weight divided by height squared (kg/m^2^). All the analyses above were calculated in R software.

#### 2.6.2 Discriminant analyses for patients and HCs based on lipidomics data

Preprocessed lipids were further analysed using the “mixOmics” (version 6.16.3) package in R software (Rohart et al., 2017). The data were centred on zero mean and unit variance (auto scaling). Principal component analysis (PCA) was used to check the homogeneity of the samples and determine whether QC samples were tightly clustered together. After removing the outliers, we developed a sparse partial least square-discriminant analysis (sPLS-DA) model, a supervised machine learning analysis, to identify the lipids that contributed most to the classification of psychiatric and HC groups. Parameter tuning processes were performed using the *tune* function to determine the optimal parameters. The performance of the tuning sPLS-DA model obtained with a balanced error rate (BER) was estimated with 7-fold cross validation and repeated 1,000 times. The optimal parameters, including the number of components and variables, were selected when the tuning model had a low classification error rate. The performance of the optimal sPLS-DA model was estimated by using the *perf* function, with 7-fold cross validation repeated 1,000 times. Evaluated indexes included BER and overall classification error rate (prediction distances were calculated by max, centroids, and Mahalanobis distance) and areas under the receiver operating curve (AUCs). We also performed univariate analysis and two-sample Wilcoxon rank-sum tests to complement the multivariate analysis, followed by false discovery rate (FDR) adjustment. The most important variables (lipids) for differentiating the psychiatric and HC groups satisfied the following cut-off criteria: 1) AUCs of the sPLS-DA model >0.8, 2) variable importance in projection (VIP) scores >1, 3) occurrence frequency of the lipids >0.8 after performing 1,000 times of cross validation, and 4) *p* value <0.05 after FDR adjustment. A total of 10 significantly altered lipids met all the above criteria to differentiate between patients and HCs.

#### 2.6.3 Identifying lipid-based subtypes utilizing consensus cluster

We developed an unsupervised cluster model to investigate the potential subtypes within the group of psychiatric patients using the data of 10 identified lipids. Consensus partitioning was performed and summarized by the “cola” package (Gu et al., 2021). Features for consensus partitioning were calculated by four methods: standard deviation (SD), median absolute deviation (MAD), coefficient of variation (CV), and ability to correlate to other rows (ATC). Partitioning methods included hierarchical clustering (hclust), k-means clustering (kmeans), partitioning around medoids (pam), and spherical k-means clustering (skmeans). The partitioning step was repeatedly executed 50 times for each partitioning method. The mean silhouette score and concordance were calculated to evaluate the cluster models and select the optimal number (k) of subtypes. The SD-skmeans model generated an optimal k of 2.

#### 2.6.4 The differences between lipid-based subtypes across multiple-level data

We compared the differences between the two subtypes in terms of clinical features (including the global functional impairment measured by GAF scale scores; symptoms severity measured by PANSS scores, YMRS scores, HAMA scores, and HAMD scores in patients as appropriate; onset age; TDP; CDP; and DUP), and brain WM microstructural alterations (measured by FA and RD) in R software. A two-sample *t* test was performed to compare the difference in GAF scores and ROI-based FA and RD data between the subtypes (followed by FDR adjustment). The 48 WM regional FA and RD effect sizes of subtypes (Cohen’s d) were also calculated.

## 3 Results

### 3.1 Demographic characteristics

We removed 7 individuals (3 patients and 4 HCs) after the lipidomic data quality control process (Supplementary Figure S1 **)**. The demographic characteristics of the remaining 346 psychiatric patients and 194 HCs are described in Table 1. There were no significant differences between patients and HCs in terms of age, sex, or BMI. The mean educational attainment years of participants in the HC group were higher than those of participants in the psychiatric group (*p* < 0.001).

**TABLE 1:** Comparison of demographic characteristics between the psychiatric patient and healthy control groups.

Variables	Patients	HC	*x* ^*2*^ */t*-statistic	*p value*
	(*n* = 346)	(*n* = 194)		
Sex^a^ (male/female)	141/205	66/128	2.11	0.15
Age^b^	24.86 ± 8.32	25.22 ± 8.22	0.48	0.63
Educational Attainment^b^ (years)	13.29 ± 2.79	15.20 ± 2.44	8.27	<0.001^***^
BMI^b^	21.04 ± 3.01	20.93 ± 2.60	−0.42	0.68

a The *p value* was obtained by the chi-square test.

b The *p value* was obtained by the two-sample *t* test.

*p < 0.05; **p < 0.01; ***p < 0.001.

Age, sex and BMI data are presented as the mean ± standard deviation. BMI was calculated as weight divided by height squared (kg/m^2^).

HC, healthy control; BMI, body mass index.

### 3.2 Discriminant analyses for patients and HCs

#### 3.2.1 Choosing optimal parameters from the tuning model

The performance of tuning the sPLS-DA model is displayed inFigure 1A. The balanced classification error rates were decreased when more components were added to the model. In the tuning model, the first two components (composed of 2 lipid features selected from the first component and 20 lipid features selected from the second component) were sufficient to achieve good performance (error rate = 0.046 ± 0.005, 7-fold cross validation repeated 1,000 times).

**FIGURE 1:**
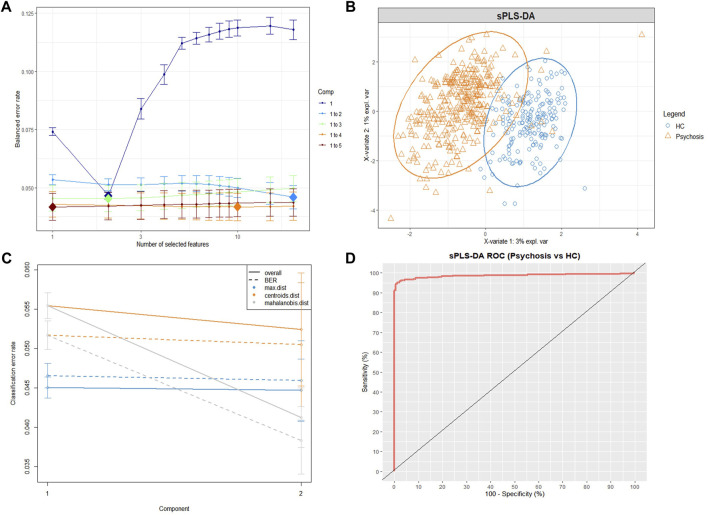
The sPLS-DA model for differentiating psychiatric patients and healthy controls using lipidomic data. **(A)** Balanced error rates (BERs) decreased when more components were added to the tuning sPLS-DA model. Here, the first 2 components (light blue line) were sufficient to achieve good performance (error rate = 0.046 ± 0.005, 1,000×7-fold cross-validation), and the optimal features of each component are indicated as a diamond plot. **(B)** The sPLS-DA sample plot with ellipse circles indicating the 95% confidence interval. The first two components of the sPLS-DA model differentiating the psychiatric patient group (orange triangle) from the HC group (blue circle). **(C)** Classification performance per component (overall and BER) for three prediction distances using repeated cross-validation (1,000×7-fold). All the classification error rates were lower than 0.06. **(D)** The ROC curve of the sPLS-DA model, and the AUC = 0.986. *sPLS-DA*, sparse partial least squares discriminant analysis; *ROC*, receiver operating curve; *AUC*, area under the receiver operating curve; *HC*, healthy control group; *Psychosis*, psychiatric patient group.

#### 3.2.2 Identifying contributing lipids for the classification of psychiatric patients and HCs

Figure 1B displays the sPLS-DA sample plot. The first two components accurately distinguished psychiatric patients from HCs. Figure 1C displays the BER and overall error rates of the two components for three prediction distances (7-fold cross validation, repeated 1,000 times). All classification error rates were less than 0.06 (details in Supplementary Table S1 **)**. The receiver operating curve (ROC), as an additional measure that helped reflect the performance of the sPLS-DA model, is depicted in Figure 1D, and the AUC = 0.986. The low classification error rates and high AUC indicate that the previous tuning process led to a final sPLS-DA model that achieved good performance. According to the cut-off criteria mentioned in the methods, 10 lipids were selected (Table 2).

**TABLE 2:** Identified differential lipids for classifying psychiatric patients and healthy controls.

Lipids	Classification	Formula	Molecular weight	VIP		Freq	Trend^a^
				Comp 1	Comp 2		
9,12-Octadecadienal	Fatty acyls/Fatty aldehydes	C_18_H_32_O	264.2455	33.38	31.50	1.00	↑^***^
20-oxo-22,23,24,25,26,27-hexanorvitamin D3	Sterol lipids/Vitamin D3 like derivatives	C_21_H_30_O_2_	314.2248	6.92	6.53	1.00	↓^***^
10-nitro-9Z,12Z-octadecadienoic acid	Fatty acyls/Nitro fatty acids	C_18_H_31_NO_4_	325.2255	0.00	9.52	1.00	↓^***^
DGTS 16:0/18:1	Other	C_44_H_83_NO_7_	737.6169	0.00	2.71	1.00	↑^***^
4-amino-3-methylbutanoic acid	γ-Aminobutyric acid analogue	C_5_H_11_NO_2_	117.0791	0.00	2.07	0.93	↓^***^
Cyclopentaneoctanoic acid	Fatty acyls/Unsaturated fatty acids	C_17_H_26_O_5_	310.1781	0.00	1.70	0.94	↑^***^
OxPC 16:0-18:1+2O	Other	C_42_H_82_NO_10_P	791.5690	0.00	1.59	0.95	↑^***^
Caprylic acid	Fatty acyls/Straight chain fatty acids	C_8_H_16_O_2_	144.1152	0.00	1.37	0.97	↑^***^
Hexadecandioic acid	Fatty acyls/Dicarboxylic acids	C_16_H_30_O_4_	286.2145	0.00	1.30	0.93	↑^***^
12-Tridecynoic acid	Fatty acyls/Unsaturated fatty acids	C_13_H_22_O_2_	210.1621	0.00	1.12	0.89	↑^***^

a Up arrow (↑) indicates an upregulated trend in psychiatric patients compared with healthy controls; down arrow (↓) indicates a downregulated trend in psychiatric patients compared with healthy controls.

***p value < 0.001, adjusted by false discovery rate (FDR) adjustment.

*VIP*, variable importance in projection; *Comp1*, first component of the classification model; *Comp2*, second component of the classification model; *Freq*, lipid occurrence frequency when performing 1,000 times cross-validation; *DGTS*, diacylglyceryl- N,N,N- trimethylhomoserine; *OxPC*, [2-[(Z)-12,13-dihydroxyoctadec-9-enoyl]oxy-3-hexadecanoyloxypropyl] 2-(trimethylazaniumyl)ethyl phosphate.

### 3.3 Consensus cluster analysis within the group of psychiatric patients

**FIGURE 2:**
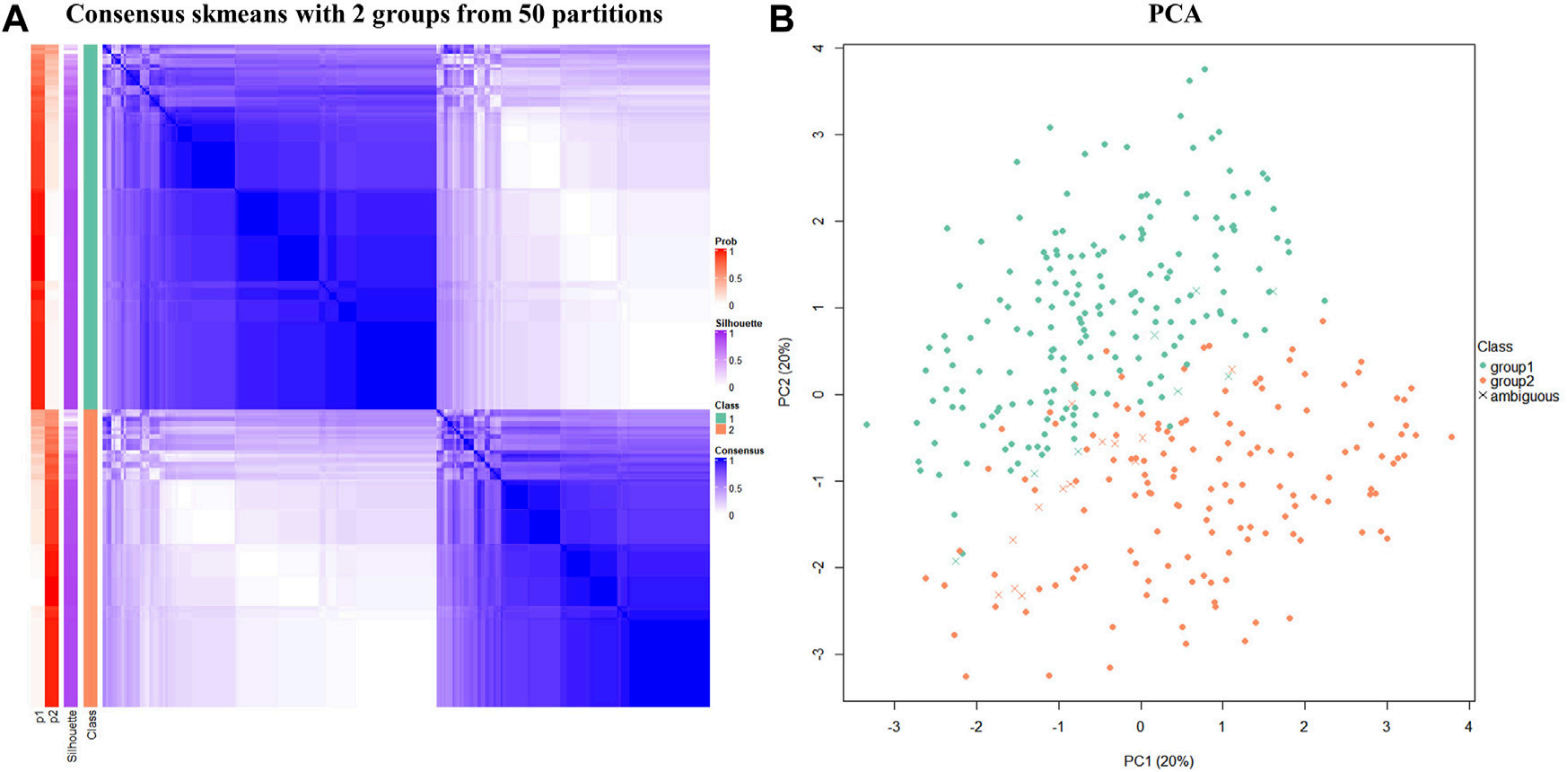
The consensus clustering analysis identified two subgroups within psychiatric patients. **(A)** The consensus matrix heatmap plot visualized the stability of the two subtypes. The “p1” and “p2” labels refer to the probability of the sample being classified into “class 1” and “class 2,” respectively, after clustering was repeated 50 times. “Prob” refers to the calculated probability of the sample being classified into the corresponding subgroup. The “consensus” legend refers to how consistent two samples were in the same subgroup. **(B)** The PCA plot confirmed that there were two subgroups of psychiatric patients. The confident samples (silhouette score >0.5) are classified into two subgroups obviously separated from each other, and the ambiguous samples (silhouette score <0.5) are indicated by crosses on the plot.

Consensus clustering was performed among the psychiatric patients. The skmeans model generated stable partitions compared to other methods, especially when combined with SD (details in Supplementary Table S2). The confident samples with silhouette scores >0.5 (N = 319) were classified into two stable subtypes named the Cluster 1 and Cluster 2 subtypes (mean silhouette = 0.8; concordance = 0.9). The consensus heatmap (Figure 2A) provides a visual representation of how consistent two samples were in the same subtype. The PCA plot (Figure 2B) also confirmed that the two subtypes were separate from each other.

### 3.4 The differences in lipid-based subtypes across multiple-level data

#### 3.4.1 Demographic characteristics of the two subtypes

There were no significant differences between the two lipid-based subtypes in terms of demographic characteristics (age, sex, educational attainment, and BMI) (Table 3).

**TABLE 3:** Comparison of demographic characteristics and functional impairment assessment between the lipid-based subgroups.

Variables	Cluster 1	Cluster 2	*x* ^*2*^ */t*-statistic	*p value*
	(*n* = 179)	(*n* = 140)		
Demographic characteristic				
Sex^a^ (male/female)	64/115	62/78	2.05	0.15
Age^b^	25.11 ± 8.45	24.21 ± 7.73	0.98	0.33
Educational Attainment^b^ (years)	13.15 ± 2.92	13.40 ± 2.60	−0.82	0.41
BMI^b^	20.82 ± 2.84	21.24 ± 3.28	−1.22	0.22
Clinical diagnosis distribution^a^				
SCZ	33	65	65.81	<0.001***
BP	59	64		
MDD	87	11		
Clinical assessment^b^	(*n* = 156)	(*n* = 123)		
GAF scale scores	54.96 ± 13.08	50.17 ± 13.83	2.94	0.0036**

a The *p* value was obtained by the chi-square test.

b The *p* value was obtained by the independent two-sample *t* test.

*p < 0.05; **p < 0.01; ***p < 0.001.

Age, sex, BMI and GAF scale scores are presented as the mean ± standard deviation. BMI was calculated as weight divided by height squared (kg/m^2^). HC, healthy control; BMI, body mass index; GAF, Global Assessment of Functioning Scale.

#### 3.4.2 The differences in clinical features of the two subtypes

**FIGURE 3:**
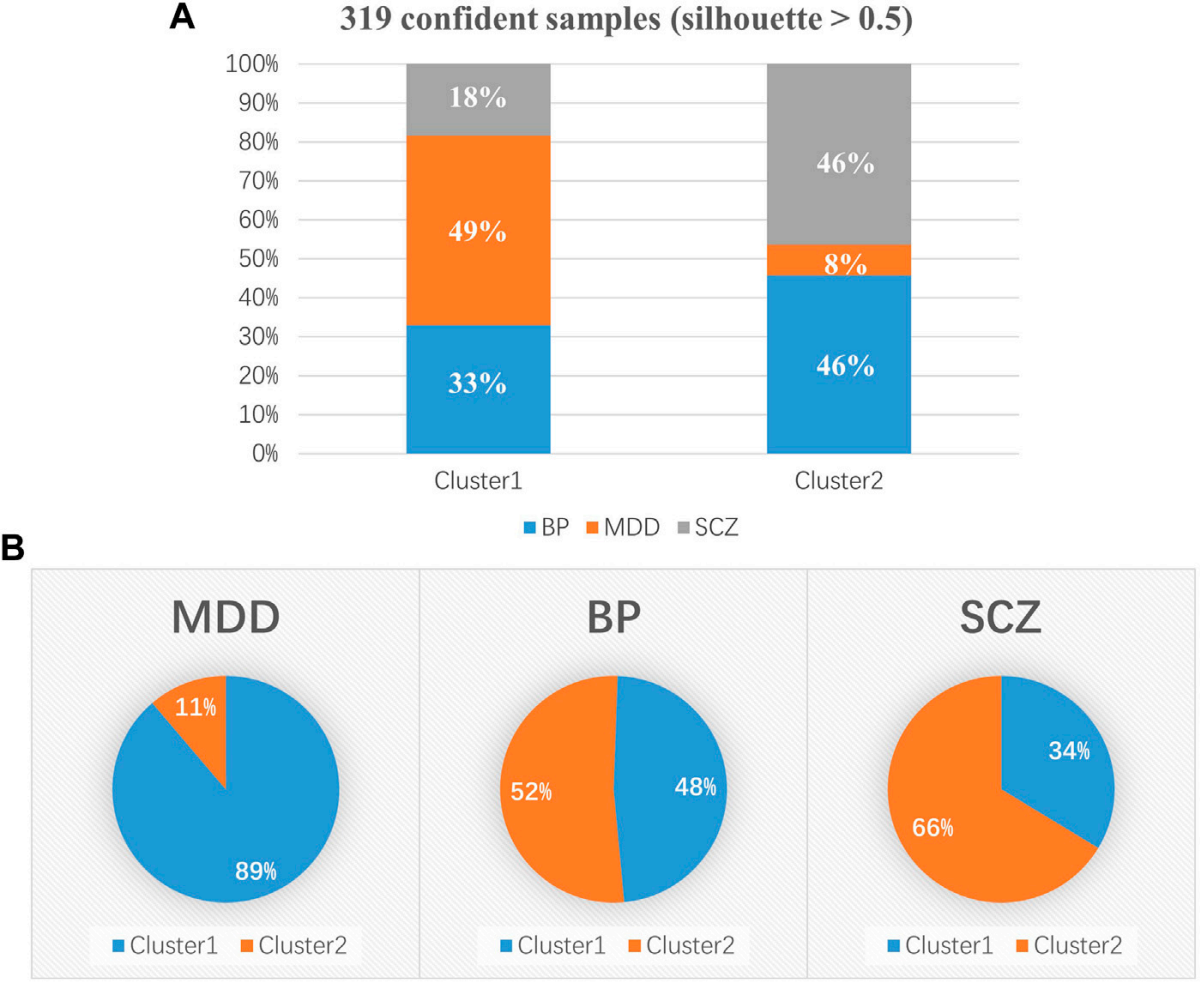
Distribution of clinical diagnoses in the two subgroups. **(A)** Cluster 1 included 179 psychiatric patients, consisting of 33 (18%) patients with SCZ, 59 (33%) patients with BP and 87 (49%) patients with MDD, and Cluster 2 included 140 psychiatric patients. **(B)** A higher proportion of MDD patients was present in Cluster 1 (89%) than in Cluster 2 (11%). There were more patients with SCZ in Cluster 2 (66%) than in Cluster 1 (34%). Patients with BP were uniformly distributed in Cluster 1 (52%) and Cluster 2 (48%). SCZ, schizophrenia; BP, bipolar disorder; MDD, depressive disorder. *p < 0.05; **p < 0.01; ***p < 0.001.

Cluster 1 included 179 patients (52.65%), and Cluster 2 included 140 patients (41.18%). The clinical diagnosis distribution varied between the two subtypes (*x* ^*2*^ = 65.81, *p* < 0.001) (Table 3). Cluster 1 consisted of 33 (18%) patients with SCZ, 59 (33%) patients with BP and 87 (49%) patients with MDD, and Cluster 2 consisted of 65 (46%) patients with SCZ, 64 (46%) patients with BP and 11 (8%) patients with MDD (Figure 3A). A higher proportion of patients with MDD was in Cluster 1 (89%) than in Cluster 2 (11%). In contrast, more patients with SCZ were allocated to Cluster 2 (66%) than to Cluster 1 (34%). Patients with BP were uniformly distributed in Cluster 1 (52%) and Cluster 2 (48%) (Figure 3B). In regard to clinical features, Cluster 2 patients (50.17 ± 13.83) showed significantly lower GAF scores than Cluster 1 patients (54.96 ± 13.08) (*t* = 2.94, *p* = 0.0036) (Table 3). The clinical features of schizophrenia did not show any difference between the two clusters (Supplementary Table S3); the distribution of bipolar I and bipolar II disorder differed in the two clusters (*x* ^*2*^ = 4.87, *p* = 0.027), and a higher proportion of bipolar I patients occurred in Cluster 2 (61%) than in Cluster 1 (39%) (Supplementary Table S4). In addition, the HAMA scores of MDD patients with a Cluster 2 (11.36 ± 5.73) subtype were significantly lower than those with a Cluster 1 subtype (16.07 ± 5.61) (*t* = 2.56, *p* = 0.024) (Supplementary Table S5). Table 4 provides a general summary schema to summarize the comparison results of the clinical features of SCZ, BP and MDD allocated to the two subtypes.

**TABLE 4:** Comparison of clinical features of SCZ, BP and MDD patients between the lipid-based subgroups.

Variables^a^	SCZ	BP	MDD
PANSS scale	−		
YMRS scale		−	
HAMA scale		−	+
HAMD scale		−	−
Maternal gestation	−		
Full-term/preterm pregnant period	−		
Full-term normal/caesarean delivery	−		
Bipolar I/II subtype		+	
Psychotic feature		−	
Onset age	−	−	−
TDP (month)	−	−	−
CDP (month)		−	−
DUP (month)		−	−
Current episode state		−	
Depressive episodes			−

a + indicates a significant difference in SCZ, BP, and MDD patients between the lipid-based subgroups in the corresponding item; − indicates there are no significant differences.

PANSS, positive and negative syndrome scale; YMRS, young mania rating scale; HAMA, hamilton anxiety scale; HAMD, hamilton depression scale; TDP, total duration of illness period; CDP, current duration of illness period; DUP, duration of untreated period.

#### 3.4.3 Brain white matter alterations between the two subtypes

**FIGURE 4:**
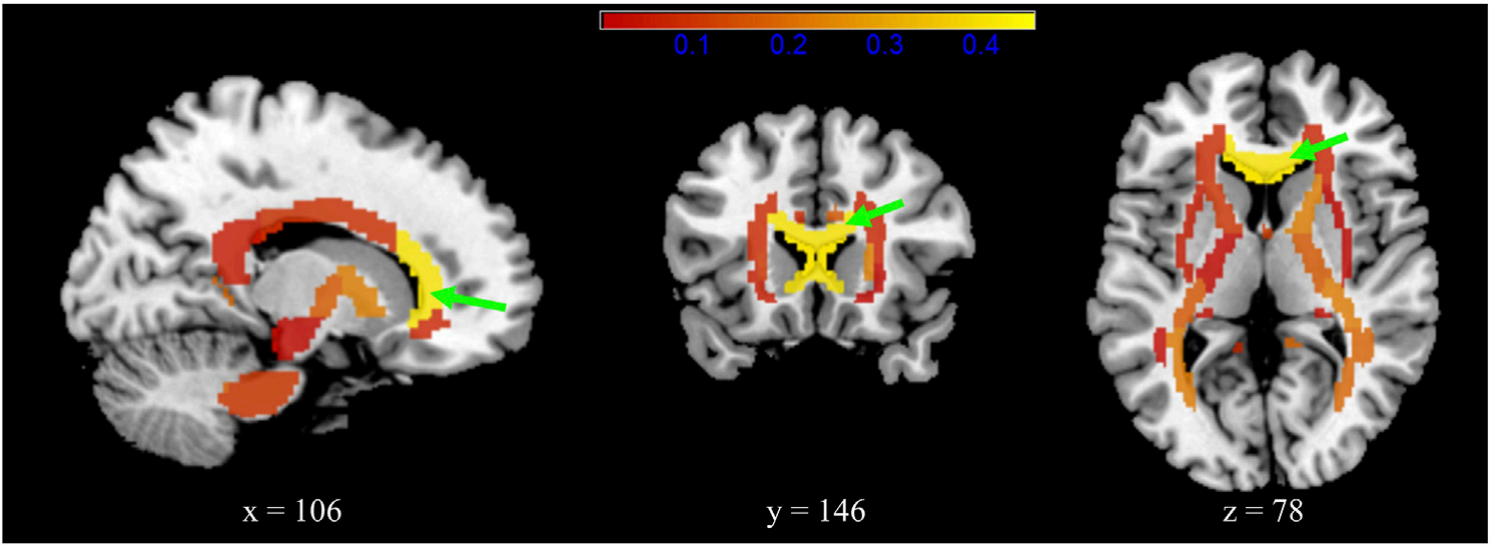
Radial diffusivity (RD) differences between the two subgroups for 48 white matter brain regions that represent the major fasciculi. The colour bar (red–yellow) indicates the mean effect size of the group (Cohen’s d). The genu of the corpus callosum (green arrow) showed significantly increased RD in patients in the Cluster 2 subgroup (Cohen’s d = 0.405; *p.adj* = 0.018).

After FDR adjustment, patients in the Cluster 2 group showed significantly increased RD (1.169 ± 0.768) compared to those in the Cluster 1 group (0.857 ± 0.771) (Cohen’s d = 0.405; *t* = −3.591; *p.adj* = 0.018), mainly along the genu of corpus callosum (GCC) (Figure 4; Supplementary Table S6). Patients within the Cluster 2 subtype showed trends of decreased FA along the fornix (including the column and body of the fornix) (*p* = 0.018, Cohen’s d = 0.266) and right posterior thalamic radiation (*p* = 0.032, Cohen’s d = 0.243) and increased FA mainly along the left hippocampus region (*p* = 0.044, Cohen’s d = 0.226) compared to patients within the Cluster 1 subtype, although significance did not survive FDR adjustment (Supplementary Table S7). Supplementary Table S8 provides the association of identified lipids and brain WM alterations in psychiatric patients.

## 4 Discussion

In this study, discriminant analysis identified 10 disease-specific lipids that contribute to the classification of psychiatric patients (including patients with SCZ, BP, and MDD) and HCs. We further found two lipid-based subtypes (named the Cluster 1 and Cluster 2 subtypes) within the psychiatric patients utilizing cluster analysis. The two subtypes differed in clinical features and brain WM abnormalities. The clinical diagnosis distribution significantly differed in the two subtypes: patients with BP were uniformly distributed in the two subtypes, but a higher proportion of patients with MDD (89%) was noted in Cluster 1, and a higher proportion of patients with SCZ (66%) was noted in Cluster 2. Patients in Cluster 2 showed significantly lower GAF scores than those in Cluster 1. Moreover, the patients within Cluster 2 showed significantly increased RD in the GCC, decreased FA trends in the fornix and posterior thalamic radiation, and increased FA trend in the hippocampus.

Patients within the Cluster 2 subtype mainly consisted of those with SCZ and BP (a total of 92%). Previous studies have indicated that SCZ and BP are characterized by similar biological patterns, such as high genetic correlation (Cross-Disorder Group of the Psychiatric Genomics et al., 2013) and comparable WM abnormalities. A large-scale meta-analysis has reported that patients with SCZ/BP (but not in MDD) shared limbic system (such as the fornix) abnormalities (Koshiyama et al., 2020), and posterior thalamic contraction (Mamah et al., 2016). In this study, the interesting constituent ratio of clinical diagnosis, and the decreased FA trend along the fornix and posterior thalamic region in patients within the Cluster 2 subtype were supported and consistent with previous findings. We also observed that bipolar I and bipolar II disorder distributions differed between the two subtypes. This finding is consistent with previous studies that found biological heterogeneity between bipolar I and bipolar II disorder (Charney et al., 2017; Huang et al., 2022). HAMA scores of MDD patients differed in the two subtypes. However, considering that only 11 MDD patients were allocated to Cluster 2, the small sample size may not satisfy the statistical power. Other clinical features of SCZ, BP and MDD showed no differences between the two lipid-based subtypes, which support that there are mismatch boundaries between biological subtypes and clinical diagnosis based on descriptive data. The differential findings of the two subtypes support the high similarity among SCZ and BP patients.

In addition to the lipid-based biological pattern, Cluster 2 patients also presented differential WM abnormalities measured by RD mainly along the GCC region and lower GAF scores. Brain WM abnormalities in the corpus callosum have been widely and consistently reported across several psychiatric disorders by meta-analyses, especially in the GCC of patients with SCZ (Kelly et al., 2018; Koshiyama et al., 2018; Favre et al., 2019; van Velzen et al., 2020). Lower GAF scores indicate severer psychological, social and occupational functioning impairment. The GCC is the bend of the anterior corpus callosum; thus, it facilitates prefrontal interhemispheric connectivity and relates to social competence, planning and memory performance, etc. (Paul et al., 2007). We speculate that the greater functioning impairment of patients within the Cluster 2 subtype are potentially the consequences of structural abnormalities in the GCC. As RD is a specific index reflecting the demyelination or morphology abnormalities of fibre tracts (Song et al., 2002; Song et al., 2005), the significantly elevated RD in the GCC region of Cluster 2 patients might reflect greater brain WM lesions here. In summary, these findings indicate that the lipid-based subtypes across psychiatric disorders also showed differential multiple-level biological characteristics.

The 10 identified lipids from the sPLS-DA model relate to several abnormal physiological processes, including inflammation and oxidative stress, brain structural or functional abnormality regulation, and metabolic deterioration. 10-Nitro-9Z,12Z-octadecadienoic acid (nitrolinoleic acid, LNO_2_) is rich in human plasma and red cell membranes. It acts as a lipid-derived mediator in activating antioxidant signalling pathways (Kalyanaraman, 2004; Koutoulogenis and Kokotos, 2021). LNO_2_ also exhibits robust cell signalling activities as an anti-inflammatory (Coles et al., 2002; Schopfer et al., 2005; Wright et al., 2006; Koutoulogenis and Kokotos, 2021). In this study, decreased plasma LNO_2_ might indicate the vulnerable anti-inflammatory status of psychiatric patients. In addition, there are several other identified lipids associated with inflammation and oxidative stress. Of note, change in hexadecanedioic acid level was reported to be related to inflammatory status, and it contributed to the classification of SCZ and HCs in previous studies (Cui et al., 2020; Qian et al., 2021). The diacylglyceryl-N,N,N-trimethylhomoserine (DGTS)16:0/18:1 level is considered a biomarker reflecting low oxidative stability among wheat varieties (Wei et al., 2021). A meta-analysis has revealed that first-episode psychiatric patients exhibited a proinflammatory and vulnerable antioxidant status (Fraguas et al., 2019). Recently, a proposed hypothesis illuminated immune/inflammatory-mediated alteration of brain WM in the limbic system as the main pathophysiological mechanism of psychiatric disorders (Magioncalda and Martino, 2022). In this study, DGTS 16:0/18:1 and hexadecandioic acid positively related to RD of GCC and FA of left hippocampus. These above-altered lipids might indicate inflammation and oxidative stress imbalance in psychiatric patients.

Although there is insufficient evidence, previous studies have implicated that the altered 12-tridecynoic acid and 4-amino-3-methylbutanoic acid levels might be related to the regulation of brain structural abnormalities. The Wnt signalling pathways are important in modulating synapse growth and synaptic plasticity in humans, and altered Wnt signalling was documented in patients with SCZ and BP (Tabares-Seisdedos and Rubenstein, 2009; Hoseth et al., 2018). 12-Tridecynoic acid is one of the lipids that participates in the fatty acylation/deacylation of Wnt proteins (Gao and Hannoush, 2014; Torres et al., 2019), which are necessary for their biofunction (Willert et al., 2003; Rios-Esteves et al., 2014). However, there is no direct evidence linking the changes in fatty acid levels to Wnt protein activation. In this study, the effect of increased 12-tridecynoic acid levels on the brain structure is unknown, and further studies could perhaps investigate the relationship between them by evaluating Wnt signalling pathways. 4-Amino-3-methylbutanoic acid is a 3-substituted γ-aminobutyric acid (GABA) analogue with greater affinity for GABA receptors in the human brain (Nicholson et al., 1979). Moreover, it could raise GABA levels by increasing L-glutamic acid decarboxylase (GAD) activity in the mouse brain and produce an anticonvulsant effect (Silverman et al., 1991; Taylor et al., 1992). However, there is also no evidence linking decreased 4-amino-3-methylbutanoic acid concentrations with the function of central GABAergic neurons, which requires more research. These peripheral lipid alterations may provide clues and broaden our understanding of the mechanisms underlying brain structure abnormalities, which is one of the main pathogenic mechanisms of psychiatric disorders.

Unhealthy dietary and behaviour patterns have recently been noted as risk factors for the metabolic deterioration of patients with SCZ, BP, and MDD (Beyer and Payne, 2016; Vancampfort et al., 2017). A previous randomized crossover trial reported that the high fiber consumption dietary intervention decreased plasma 9,12-octadecadienal level. It is considered to be involved in mediating the positive effect of a healthy diet on maintaining satiety and preventing obesity (Lankinen et al., 2011). In this study, increased 9,12-octadecadienal level in psychiatric patients might reflect the unhealthy dietary pattern (such as low consumption of fiber and fruit) of these patients (Dipasquale et al., 2013). Caprylic acid is important in regulating food intake behaviour by esterifying ghrelin, which is a key peptide hormone with orexigenic biofunction (Kojima et al., 1999; Kojima and Kangawa, 2002; Delporte, 2013). Previous randomized controlled trial studies have reported that ingestion of caprylic acid helps stimulate food intake behaviour, and has been used to treat anorexia nervosa (Kawai et al., 2017) and cachectic patients (Ashitani et al., 2009). Disordered eating behaviours are common among SCZ, BP (such as binge eating, food cravings, and night eating), and MDD (emotional and external eating) patients, which were occurred in the initial onset and cannot all be attributed to the side effects of drug treatment (Paans et al., 2018; Stogios et al., 2020; Sankaranarayanan et al., 2021). In this study, the increased caprylic acid level might provide clues about the disordered eating behaviour among psychiatric patients. Apart from attention to dietary patterns and disordered eating behaviour, an unhealthy behaviour pattern is another important risk factor for metabolic deterioration. Of note, 20-oxo-22,23,24,25,26,27-hexanorvitamin D3 is only synthesized by skin tissue in humans through ultraviolet B (UVB) induced physicochemical processes (Slominski et al., 2012). Since UVB is essential in the synthetic process, it is reasonable to infer that the significantly decreased 20-oxo-22,23,24,25,26,27-hexanorvitamin D3 levels in psychiatric patients may be attributed to lower sunlight exposure, which is associated with unhealthy behavioural patterns (such as sedentary behaviour). A previous meta-analysis reported that patients with SCZ, BP and MDD have significant sedentary behaviour (average 476 min per day) during waking hours and low activity (38.4 min per day) (Vancampfort et al., 2017). Above all, the alterations in 9,12-octadecadienal, caprylic acid, and 20-oxo-22,23,24,25,26,27-hexanorvitamin D3 levels might reflect the unhealthy dietary and behavioural pattern of the mechanism that underlying metabolic deterioration of psychiatric patients.

There are some limitations to this study. First, as antipsychotic drugs affect lipid metabolism, we tried our best to recruit drug-naïve patients. In this study, all the recruited schizophrenia patients were first-episode and drug-naïve, however, drug-naïve bipolar disorder patients were hard to recruit due to the diagnosis delay and high misdiagnosis rate (Culpepper, 2014; Fritz et al., 2017). We recruited 63 (47.8%) drug-naïve BP patients and set at least 2 weeks wash-out period for other BP patients (the median current duration of illness period was 2 months). When it comes to MDD patients, the main treatment strategies are SSRI/SNRI drugs, even so, we recruited 77 (77.3%) drug-naïve patients and also set at least 2 weeks wash-out period for other MDD patients. Then, because sample collection at a single center with lower variability may restrict the generalization of these findings. We will conduct independent sample validation in the future study to make these findings more robust and convincing. Moreover, the biological functions of some identified lipids are attractive, such as LNO_2_ and caprylic acid. Although previous studies have evidenced their biological function in psychiatric patients, further studies could better elucidate the effects of these lipids on psychiatric diseases. For example, adding inflammatory factors examination, and the questionnaire about the dietary and behaviour patterns.

## 5 Conclusion

In conclusion, our findings suggested that peripheral blood lipidomic profile alterations could help identify homogeneous transdiagnostic subtypes across psychiatric disorders consisting of SCZ, BP and MDD. One of the subtypes that mainly consisted of patients with SCZ and BP represented more severe brain WM abnormalities and functional impairments. It is suggested that lipid-based subtypes might help identify patients with differential biological characterizations.

## Data Availability

The datasets presented in this article are not readily available because According to the Regulations of the People’s Republic of China on the Administration of Human Genetic Resources, which came into effect on 1 July 2019, those who provide or open to use human genetic resources information to foreign organizations, individuals and institutions established or actually controlled by them shall submit the information to the administrative department of science and technology under The State Council for the record and submit backup information. The plasma sample analyzed in this article are also within the limits of this regulation. Requests to access the datasets should be directed to litaozjusc@zju.edu.cn.
