# Supplementary material for: Identifying transdiagnostic biological subtypes across schizophrenia, bipolar disorder, and major depressive disorder based on lipidomics profiles
Source: Front Cell Dev Biol. 2022 Sep 5;10:969575. doi: 10.3389/fcell.2022.969575 (PMC9483200; doi:10.3389/fcell.2022.969575)
Supplement: Supplementary file 1 [file DataSheet1.docx]

Supplementary Material

# Supplementary Method

## Blood sample collection and lipid extraction

The samples were centrifuged at 2000 × g for 5 min at 4 ℃, and then the upper layer (plasma) was transferred to a cryovial and stored at -80 ℃ for further processing. The plasma samples were delivered to Novogene Co., Ltd. (Beijing, China) in boxes with solid carbon dioxide for lipid extraction and UHPLC–MS/MS analyses. Plasma samples were thawed at 4 ℃, and 100 μL samples were transferred into glass tubes with a Teflon-lined cap. Then, 0.75 ml methanol was added, and the tubes were vortexed. Then, 2.5 ml of MTBE was added to the mixture and incubated for 1 hour at room temperature on a shaker. Next, 0.625 ml of MS-grade water was added to induce phase separation. Upon 10 min of incubation at room temperature, the samples were centrifuged at 1,000 × g for 10 min. The upper (organic) phase was collected, and then the lower phase was re-extracted with 1 mL of the solvent mixture (MTBE/methanol/water (10:3:2.5, v/v/v)) to collect the upper phase again. The organic phases were dried and dissolved in 100 μL of isopropanol for storage. Quality control samples were prepared by pooling the same volume from each sample.

## UHPLC–MS/MS analysis

UHPLC–MS/MS analyses were performed using a Vanquish UHPLC system (Thermo Fisher, Germany) coupled with an Orbitrap Q ExactiveTM HF mass spectrometer (Thermo Fisher, Germany). Samples were injected into a Thermo Accucore C30 column (150x2.1 mm, 2.6 μm) using a 20-min linear gradient at a flow rate of 0.35 mL/min with the column temperature set at 40°C. Mobile phase buffer A was acetonitrile/water (6/4) with 10 mM ammonium acetate and 0.1% formic acid; buffer B was acetonitrile/isopropanol (1/9) with 10 mM ammonium acetate and 0.1% formic acid. The solvent gradient was set as follows: 30% B, initial; 30% B, 2 min; 43% B, 5 min; 55% B, 5.1 min; 70% B, 11 min; 99% B, 16 min; and 30% B, 18.1 min. The Q ExactiveTM HF mass spectrometer was operated in positive [negative] polarity mode with sheath gas: 20 arbitrary units, sweep gas: 1 arbitrary unit, auxiliary gas rate: 5 [7], spray voltage: 3 kV, capillary temperature: 350°C, heater temperature: 400°C, S-Lens RF level: 50, resolving power (full scan): 120000, scan range: 114–1700 m/z, automatic gain control target: 1e6, resolving power (MS^2^): 30000 (Top20), normalized collision energy: 25; 30 [20; 24; 28], injection time: 100 ms, isolation window: 1 m/z, automatic gain control target (MS^2^): 1e5, and dynamic exclusion: 15 s.

## Qualitative and quantitative lipid identification

The raw data files were processed using Compound Discoverer 3.01 (Thermo Fisher) to perform peak alignment, peak picking, quantitation, and imputation for each metabolite. The main parameters were set as follows: retention time tolerance, 0.2 minutes; actual mass tolerance, 5 ppm; signal intensity tolerance, 30%; signal/noise ratio, 3; and minimum intensity, 100000. After that, the data were used to predict the molecular formula based on additive ions, molecular ion peaks and fragment ions. Then, peaks were matched with the Lipidmaps or Lipidblast database for metabolite identification, and accurate qualitative and relative quantitative results were obtained.

## MRI scan data acquisition

All participants underwent MRI scans within 3 days of enrolment using a Philips 3.0 T (Achieva, Amsterdam, the Netherlands) scanner equipped with an eight-channel head coil. Diffusion magnetic resonance imaging (dMRI) data were acquired using an echo-planar imaging (EPI) sequence with parameters with 2 diffusion gradient directions, b-values 0 and 1,000 mm^−2^, echo time (TE) = 92 ms, repetition time (TR) = 10 407 ms, FOV (field of view) = 256 × 256 mm^2^, acquisition matrix size = 128 × 128, voxel size = 2 × 2 × 2 mm^3^, EPI factor = 67, slice thickness = 2.0 mm, SENSE factor 2 in the anterior-posterior direction, 75 slices throughout the whole brain, and strong fat suppression. T1w images were acquired by a magnificent-prepared rapid gradient-echo sequence with the following parameters: repetition time (TR) = 8.1 ms, echo time (TE) = 3.7 ms, flip angle = 7°, slice thickness = 1 mm (no slice gap), 188 sagittal slices, matrix size: 256 × 256, field of view (FOV): 256 × 256 mm^2^, and voxel size: 1 × 1 × 1 mm^3^. All scans were reviewed by an experienced neuroradiologist to exclude brain organic abnormalities.

### Lipidomic data preprocessing and quality control.

After removing metabolic features with RSD > 20% and QC-RLSC signal correction for positive **(Figure S1 A)** and negative polarity modes **(Figure S1 C)**, all QC samples were clustered tightly on PCA plots, indicating satisfactory similarities. We excluded 7 outliers after checking the homogeneity of the samples. The other PCA plots showed the sample distribution **(Figure S1 B, D)** after log2 transformation, batch correction and data filtering. A total of 1164 lipids remained for discrimination analysis.

# Supplementary Figures and Tables

## Supplementary Figures


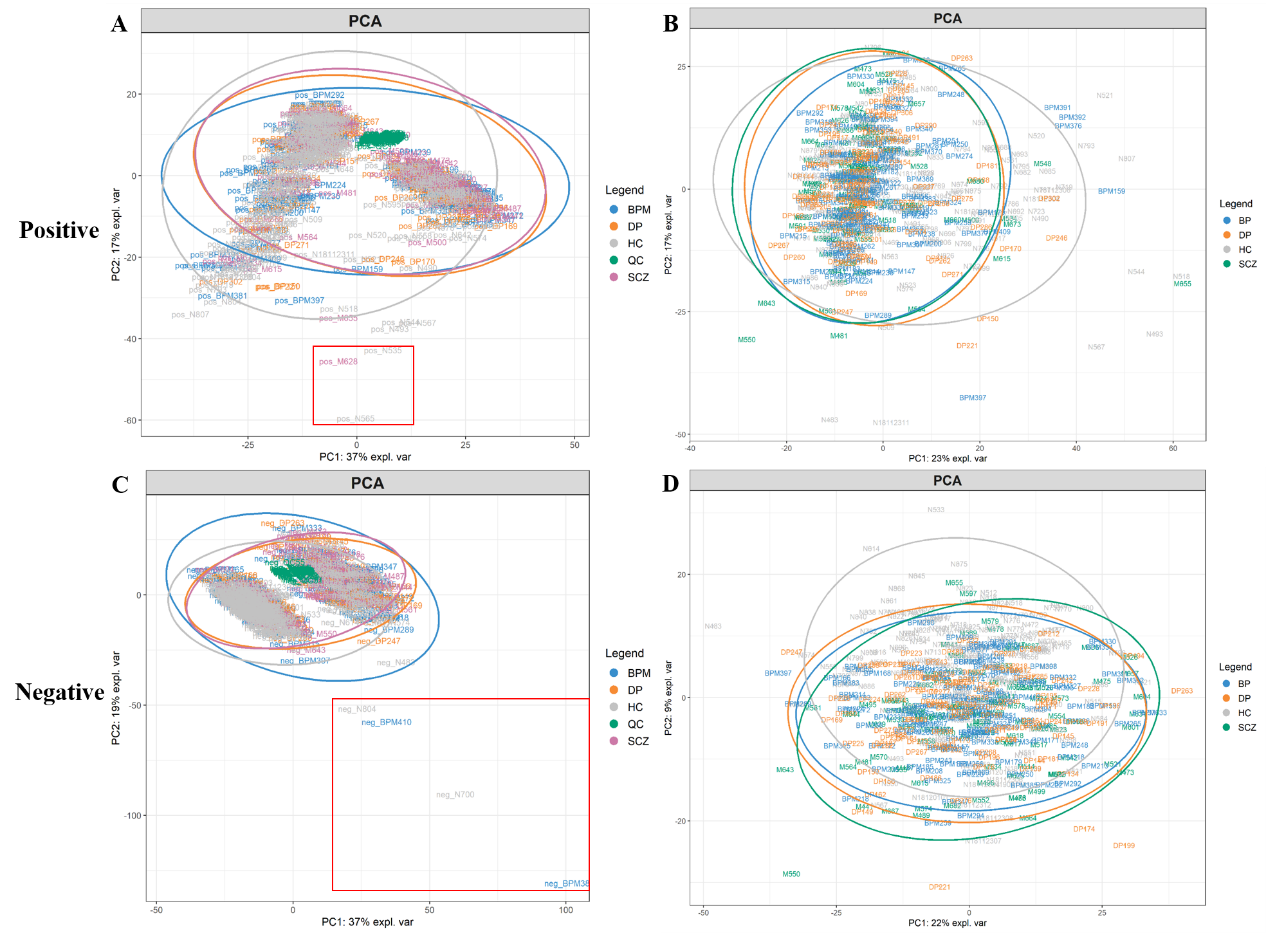


**Supplementary Figure 1.** PCA plots show the sample distributions. The red square indicates 7 outliers. (A) The clustering of the QC samples (green dots) tightly clustered together for positive polarity mode after removing points with RSD >20% and QC-RLSC signal correction. (B) Sample distribution after removing outliers, log2 transformed and batch corrected for positive polarity mode. (C) The clustering of the QC samples (green dots) tightly clustered together for negative polarity mode after removing points with RSD >20% and QC-RLSC signal correction. (D) Sample distribution after removing outliers, log2 transformed and batch corrected for negative polarity mode. The ellipse circles indicate the 95% confidence interval.

## Supplementary Tables

**Table S1. The classification error rate of each component in the sPLS-DA model**

| **Variable** | **Max** | **Centroids** | **Mahalanobis** |
| --- | --- | --- | --- |
| Overall classification error rate | | | |
| Component 1 | 0.045 | 0.055 | 0.055 |
| Component 2 | 0.045 | 0.052 | 0.041 |
| Balanced error rate | | | |
| Component 1 | 0.047 | 0.052 | 0.052 |
| Component 2 | 0.046 | 0.050 | 0.038 |

The classification error rate of each component measured by max, centroid and Mahalanobis distance

**Table S2. The mean silhouette and concordance of each cluster model**

| **Cluster model** | Mean silhouette | | | | Concordance | | | |
| --- | --- | --- | --- | --- | --- | --- | --- | --- |
|  | hclust | kmeans | pam | skmeans | hclust | kmeans | pam | skmeans |
| k = 2 | | | | | | | | |
| SD | 0.41 | 0.38 | 0.64 | 0.80 | 0.72 | 0.75 | 0.80 | 0.90 |
| MAD | 0.46 | 0.59 | 0.67 | 0.72 | 0.71 | 0.76 | 0.80 | 0.86 |
| ATC | 0.48 | 0.41 | 0.60 | 0.78 | 0.74 | 0.74 | 0.78 | 0.88 |
| CV | 0.48 | 0.54 | 0.65 | 0.73 | 0.74 | 0.75 | 0.80 | 0.86 |
|  |  |  |  |  |  |  |  |  |
| k = 3 | | | | | | | | |
| SD | 0.41 | 0.38 | 0.64 | 0.61 | 0.72 | 0.75 | 0.80 | 0.90 |
| MAD | 0.46 | 0.59 | 0.67 | 0.57 | 0.71 | 0.76 | 0.80 | 0.86 |
| ATC | 0.48 | 0.41 | 0.60 | 0.62 | 0.74 | 0.74 | 0.78 | 0.88 |
| CV | 0.48 | 0.54 | 0.65 | 0.62 | 0.74 | 0.75 | 0.80 | 0.86 |
|  |  |  |  |  |  |  |  |  |
| k = 4 | | | | | | | | |
| SD | 0.41 | 0.52 | 0.42 | 0.28 | 0.66 | 0.73 | 0.65 | 0.42 |
| MAD | 0.40 | 0.42 | 0.38 | 0.38 | 0.63 | 0.68 | 0.63 | 0.62 |
| ATC | 0.44 | 0.46 | 0.40 | 0.41 | 0.67 | 0.68 | 0.68 | 0.70 |
| CV | 0.34 | 0.49 | 0.37 | 0.43 | 0.61 | 0.70 | 0.60 | 0.69 |
|  |  |  |  |  |  |  |  |  |
| k = 5 | | | | | | | | |
| SD | 0.30 | 0.41 | 0.33 | 0.48 | 0.59 | 0.66 | 0.55 | 0.73 |
| MAD | 0.30 | 0.40 | 0.31 | 0.47 | 0.54 | 0.65 | 0.58 | 0.71 |
| ATC | 0.32 | 0.45 | 0.29 | 0.45 | 0.56 | 0.65 | 0.55 | 0.65 |
| CV | 0.25 | 0.41 | 0.34 | 0.45 | 0.53 | 0.66 | 0.56 | 0.67 |
|  |  |  |  |  |  |  |  |  |
| k = 6 | | | | | | | | |
| SD | 0.24 | 0.41 | 0.34 | 0.51 | 0.56 | 0.61 | 0.56 | 0.70 |
| MAD | 0.21 | 0.45 | 0.33 | 0.52 | 0.50 | 0.63 | 0.63 | 0.73 |
| ATC | 0.23 | 0.40 | 0.33 | 0.52 | 0.50 | 0.64 | 0.51 | 0.74 |
| CV | 0.29 | 0.42 | 0.28 | 0.48 | 0.53 | 0.63 | 0.46 | 0.63 |

Four consensus partitioning methods were combined with four methods to calculate the features.

*k,* the selected optimal number of subgroups of the cluster model; *hclust*, hierarchical clustering; *kmeans*, k-means clustering; *pam*, partitioning around medoids; *skmeans*, spherical k-means clustering. *SD*, standard deviation; *MAD*, median absolute deviation; *CV*, coefficient of variation; *ATC*, ability to correlate to other rows.

Table S3. The clinical features of lipid-based schizophrenia biological subtypes

| **Variables** | **Cluster 1** | **Cluster 2** | ***x^2^/* *t*-statistic** | ***p value*** |
| --- | --- | --- | --- | --- |
|  | **（n = 33）** | **（n = 65）** |  |  |
| **Severity of clinical symptoms** | |  |  |  |
| PANSS scale score ^a^ | (n = 29) | (n = 56) |  |  |
| Total score | 91 ± 19.63 | 83.86 ± 19.64 | 1.59 | 0.12 |
| Positive | 23.24 ± 6.43 | 21.52 ± 6.04 | 1.19 | 0.24 |
| Negative | 23.79 ± 6.53 | 22.14 ± 8.81 | 0.98 | 0.33 |
| General | 43.97 ± 10.77 | 40.19 ± 10.56 | 1.54 | 0.13 |
|  |  |  |  |  |
| **Other clinical features** |  |  |  |  |
| Onset age ^a^ | (n = 32) | (n = 58) |  |  |
|  | 22.03 ± 7.30 | 21.67 ± 6.09 | 0.24 | 0.81 |
|  |  |  |  |  |
| With maternal gestation period illness ^b^ | 3 | 1 |  |  |
| Without maternal gestation period illness | 29 | 59 |  |  |
|  |  |  |  |  |
| Full-term pregnant period ^b^ | 31 | 59 | 1.05 | 1 |
| Preterm pregnant period | 1 | 2 |  |  |
|  |  |  |  |  |
| Full-term normal delivery ^b^ | 31 | 59 | 1.05 | 1 |
| Caesarean delivered | 1 | 2 |  |  |
|  |  |  |  |  |
| DUP (month) ^c^ | (n = 31) | (n = 53) |  |  |
| Median | 5 | 6 | 729 | 0.39 |
| The lower quartile | 1 | 2 |  |  |
| The upper quartile | 17 | 12 |  |  |

The PANSS scale scores are presented as the mean±standard deviation. *PANSS*, Positive and Negative Syndrome Scale; *DUP*, duration of untreated period

^a^ The *p value* was obtained by the independent two-sample *t* test

^b^ The *p value* was obtained by the Fisher’s exact test

^c^ The *p value* was obtained by the Wilcoxon rank sum test

^*^*p* < 0.05; ^**^*p* < 0.01; ^***^*p* < 0.001

Table S4. The clinical features of lipid-based bipolar disorder biological subtypes

| **Variables** | **Cluster 1** | **Cluster 2** | ***x^2^/* *t*-statistic** | ***p value*** |
| --- | --- | --- | --- | --- |
|  | **(n = 59)** | **(n = 64)** |  |  |
| **Severity of clinical symptoms** | |  |  |  |
| HAMA scale scores ^a^ | (n = 51) | (n = 57) |  |  |
|  | 9.63 ± 7.07 | 9.77 ± 7.12 | -0.11 | 0.91 |
| HAMD scale scores ^a^ | (n = 52) | (n = 57) |  |  |
|  | 10.04 ± 7.28 | 10.96 ± 7.44 | -0.66 | 0.51 |
| YMRS scale scores ^a^ | (n = 24) | (n = 24) |  |  |
| (patients whose scores were > 5) | 15.79 ± 6.91 | 17.54 ± 8.31 | -0.79 | 0.43 |
| **Other clinical features** |  |  |  |  |
| Onset age ^a^ | (n = 55) | (n = 62) |  |  |
|  | 19.42 ± 5.28 | 21.34 ± 7.68 | -1.59 | 0.11 |
| Bipolar I disorder ^b^ | 31 | 49 | 4.87 | 0.027^*^ |
| Bipolar II disorder | 21 | 12 |  |  |
| With psychotic features ^b^ | 38 | 45 | 0.003 | 0.95 |
| Without psychotic features | 16 | 17 |  |  |
| TDP (month) ^c^ | (n = 52) | (n = 59) |  |  |
| Median | 36 | 36 | 1487.5 | 0.79 |
| The lower quartile | 19.5 | 22.5 |  |  |
| The upper quartile | 60 | 60 |  |  |
| CDP (month) ^c^ | (n = 46) | (n = 56) |  |  |
| Median | 2 | 2 | 1206 | 0.57 |
| The lower quartile | 1 | 1 |  |  |
| The upper quartile | 4.75 | 5 |  |  |
| DUP (month) ^c^ | (n = 24) | (n = 31) |  |  |
| Median | 12 | 24 | 306.5 | 0.267 |
| The lower quartile | 1 | 3 |  |  |
| The upper quartile | 24 | 36 |  |  |
| Current episode state |  |  |  |  |
| Mania | 6 | 19 | - | - |
| Hypomania | 9 | 0 |  |  |
| Depressed | 19 | 39 |  |  |
| Mixed | 8 | 1 |  |  |
| Unspecified bipolar | 3 | 1 |  |  |
| Other specified bipolar | 8 | 1 |  |  |

The HAMA, HAMD and YMRS scale scores are presented as the mean±standard deviation. *HAMA*, Hamilton Anxiety Scale; *HAMD*, Hamilton Depression Scale; *YMRS*, Young Mania Rating Scale; *TDP*, total duration of illness period; *CDP*, current duration of illness period; *DUP*, duration of untreated period. ^a^ The *p value* was obtained by the independent two-sample *t* test. ^b^ The *p value* was obtained by the chi-square test. ^c^ The *p value* was obtained by the Wilcoxon rank sum test. ^*^*p* < 0.05; ^**^*p* < 0.01; ^***^*p* < 0.001

Table S5. The clinical features of lipid-based major depressive disorder biological subtypes

| **Variables** | **Cluster 1** | **Cluster 2** | ***x^2^/* *t*-statistic** | ***p value*** |
| --- | --- | --- | --- | --- |
|  | **（n = 87）** | **（n = 11）** |  |  |
| **Severity of clinical symptom** | |  |  |  |
| HAMA scale scores ^a^ | (n = 81) | (n = 11) |  |  |
|  | 16.07 ± 5.61 | 11.36 ± 5.73 | 2.56 | 0.024^*^ |
| HAMD scale scores ^a^ | (n = 81) | (n = 11) |  |  |
|  | 19.94 ± 5.84 | 19.82 ± 7.76 | 0.05 | 0.960 |
| **Other clinical features** |  |  |  |  |
| With suicidal ideation ^b^ | 67 | 5 | 0.26 | 0.051 |
| Without suicidal ideation | 17 | 5 |  |  |
|  |  |  |  |  |
| With suicidal behaviour ^b^ | 21 | 1 | 0.33 | 0.440 |
| Without suicidal behaviour | 62 | 9 |  |  |
|  |  |  |  |  |
| The number of depressive episodes ^b^ |  |  |  |  |
| Once | 41 | 7 | - | 0.897 |
| Twice | 17 | 2 |  |  |
| Three or more times | 15 | 1 |  |  |
|  |  |  |  |  |
| Onset age ^a^ | (n = 81) | (n = 11) |  |  |
|  | 24.47 ± 8.68 | 22.27 ± 5.27 | 1.18 | 0.250 |
|  |  |  |  |  |
| DUP (month) ^c^ | (n = 48) | (n = 8) |  |  |
| Median | 9.5 | 9.5 | 184.5 | 0.870 |
| The lower quartile | 4 | 5.25 |  |  |
| The upper quartile | 24 | 15 |  |  |
|  |  |  |  |  |
| TDP (month) ^c^ | (n = 82) | (n = 10) |  |  |
| Median | 12 | 6.5 | 509 | 0.220 |
| The lower quartile | 6 | 2.5 |  |  |
| The upper quartile | 36 | 16.5 |  |  |
|  |  |  |  |  |
| CDP (month) ^c^ | (n = 79) | (n = 10) |  |  |
| Median | 3 | 5 | 323 | 0.350 |
| The lower quartile | 1 | 2 |  |  |
| The upper quartile | 6 | 6.75 |  |  |

The HAMA and HAMD scale scores are presented as the mean ± standard deviation. *HAMA*, Hamilton Anxiety Scale; *HAMD*, Hamilton Depression Scale;

^a^ The *p value* was obtained by the independent two-sample *t* test

^b^ The *p value* was obtained by the Fisher’s exact test

^c^ The *p value* was obtained by the Wilcoxon rank sum test

^*^*p* < 0.05; ^**^*p* < 0.01; ^***^*p* < 0.001

**Table S6. Comparison of the mean radial diffusivity between the lipid-based subgroups**

| **ROI** | **Cluster 1** | **Cluster 2** | ***t*-statistic** | ***p value*** | ***p.adj*** | **Cohen's d** |
| --- | --- | --- | --- | --- | --- | --- |
| Middle cerebellar peduncle | 0.811 ± 0.795 | 0.882 ± 0.712 | -0.836 | 0.404 | 0.834 | 0.093 |
| Pontine crossing tract | -0.355 ± 0.396 | -0.346 ± 0.384 | -0.211 | 0.833 | 0.941 | 0.024 |
| Genu of corpus callosum | 0.857 ± 0.771 | 1.169 ± 0.768 | -3.591 | <0.001^***^ | 0.018^*^ | 0.405 |
| Body of corpus callosum | -0.335 ± 0.283 | -0.313 ± 0.247 | -0.737 | 0.461 | 0.852 | 0.082 |
| Splenium of corpus callosum | -0.69 ± 0.486 | -0.711 ± 0.439 | 0.407 | 0.685 | 0.941 | 0.045 |
| Fornix (column and body of the fornix) | 3.888 ± 1.101 | 3.998 ± 0.939 | -0.969 | 0.333 | 0.762 | 0.107 |
| Corticospinal tract R | 0.203 ± 0.884 | 0.223 ± 0.871 | -0.201 | 0.841 | 0.941 | 0.023 |
| Corticospinal tract L | 0.349 ± 1.035 | 0.355 ± 0.991 | -0.053 | 0.958 | 0.978 | 0.006 |
| Medial lemniscus R | -0.567 ± 0.524 | -0.616 ± 0.469 | 0.881 | 0.379 | 0.827 | 0.098 |
| Medial lemniscus L | -0.675 ± 0.358 | -0.687 ± 0.318 | 0.294 | 0.769 | 0.941 | 0.033 |
| Inferior cerebellar peduncle R | 0.226 ± 0.528 | 0.145 ± 0.461 | 1.464 | 0.144 | 0.520 | 0.162 |
| Inferior cerebellar peduncle L | 0.352 ± 0.624 | 0.275 ± 0.552 | 1.164 | 0.245 | 0.620 | 0.129 |
| Superior cerebellar peduncle R | 1.267 ± 0.851 | 1.255 ± 0.785 | 0.134 | 0.894 | 0.941 | 0.015 |
| Superior cerebellar peduncle L | 0.918 ± 0.792 | 0.901 ± 0.695 | 0.2 | 0.842 | 0.941 | 0.022 |
| Cerebral peduncle R | -0.888 ± 0.263 | -0.883 ± 0.243 | -0.191 | 0.849 | 0.941 | 0.021 |
| Cerebral peduncle L | -0.988 ± 0.289 | -0.925 ± 0.299 | -1.897 | 0.059 | 0.490 | 0.215 |
| Anterior limb of internal capsule R | -0.729 ± 0.225 | -0.78 ± 0.235 | 1.962 | 0.051 | 0.490 | 0.223 |
| Anterior limb of internal capsule L | -0.881 ± 0.178 | -0.897 ± 0.186 | 0.783 | 0.434 | 0.834 | 0.089 |
| Posterior limb of internal capsule R | -1.023 ± 0.209 | -1.056 ± 0.211 | 1.399 | 0.163 | 0.520 | 0.158 |
| Posterior limb of internal capsule L | -1.353 ± 0.253 | -1.35 ± 0.227 | -0.139 | 0.889 | 0.941 | 0.016 |
| Retrolenticular part of internal capsule R | -0.574 ± 0.179 | -0.614 ± 0.196 | 1.896 | 0.059 | 0.490 | 0.216 |
| Retrolenticular part of internal capsule L | -0.678 ± 0.2 | -0.708 ± 0.187 | 1.369 | 0.172 | 0.520 | 0.153 |
| Anterior corona radiata R | -0.111 ± 0.19 | -0.122 ± 0.174 | 0.551 | 0.582 | 0.931 | 0.062 |
| Anterior corona radiata L | -0.146 ± 0.192 | -0.133 ± 0.177 | -0.643 | 0.521 | 0.925 | 0.072 |
| Superior corona radiata R | -0.458 ± 0.151 | -0.482 ± 0.148 | 1.42 | 0.157 | 0.520 | 0.160 |
| Superior corona radiata L | -0.598 ± 0.156 | -0.612 ± 0.161 | 0.786 | 0.433 | 0.834 | 0.089 |

**Table S6 (continued). Comparison of the mean radial diffusivity between the lipid-based subgroups**

| **ROI** ^a^ | **Cluster 1** | **Cluster 2** | ***t*-statistic** | ***p value*** | ***p.adj*** | **Cohen's d** |
| --- | --- | --- | --- | --- | --- | --- |
| Posterior corona radiata R | -0.043 ± 0.248 | -0.055 ± 0.222 | 0.481 | 0.631 | 0.941 | 0.054 |
| Posterior corona radiata L | -0.096 ± 0.256 | -0.097 ± 0.245 | 0.028 | 0.978 | 0.978 | 0.003 |
| Posterior thalamic radiation R | -0.334 ± 0.242 | -0.293 ± 0.233 | -1.557 | 0.121 | 0.520 | 0.175 |
| Posterior thalamic radiation L | -0.126 ± 0.279 | -0.070 ± 0.247 | -1.915 | 0.056 | 0.490 | 0.213 |
| Sagittal stratum R | 0.002 ± 0.308 | -0.058 ± 0.26 | 1.87 | 0.062 | 0.490 | 0.207 |
| Sagittal stratum L | -0.011 ± 0.242 | 0.033 ± 0.266 | -1.524 | 0.129 | 0.520 | 0.174 |
| External capsule R | 0.14 ± 0.234 | 0.143 ± 0.224 | -0.124 | 0.902 | 0.941 | 0.014 |
| External capsule L | -0.007 ± 0.213 | -0.017 ± 0.195 | 0.437 | 0.662 | 0.941 | 0.049 |
| Cingulum (cingulate gyrus) R | 0.160 ± 0.37 | 0.106 ± 0.331 | 1.365 | 0.173 | 0.520 | 0.152 |
| Cingulum (cingulate gyrus) L | 0.188 ± 0.391 | 0.162 ± 0.396 | 0.583 | 0.560 | 0.928 | 0.066 |
| Cingulum (hippocampus) R | 1.156 ± 0.891 | 1.009 ± 0.782 | 1.562 | 0.119 | 0.520 | 0.173 |
| Cingulum (hippocampus) L | 1.199 ± 0.91 | 1.139 ± 0.862 | 0.606 | 0.545 | 0.928 | 0.068 |
| Fornix (cres)/Stria terminalis R | 0.43 ± 0.611 | 0.415 ± 0.572 | 0.223 | 0.823 | 0.941 | 0.025 |
| Fornix (cres)/Stria terminalis L | -0.101 ± 0.388 | -0.111 ± 0.341 | 0.245 | 0.807 | 0.941 | 0.027 |
| Superior longitudinal fasciculus R | -0.411 ± 0.185 | -0.442 ± 0.168 | 1.563 | 0.119 | 0.520 | 0.174 |
| Superior longitudinal fasciculus L | -0.435 ± 0.164 | -0.438 ± 0.182 | 0.136 | 0.892 | 0.941 | 0.016 |
| Superior fronto-occipital fasciculus R | -0.062 ± 0.367 | -0.112 ± 0.364 | 1.212 | 0.226 | 0.620 | 0.137 |
| Superior fronto-occipital fasciculus L | -0.353 ± 0.326 | -0.286 ± 0.328 | -1.809 | 0.072 | 0.490 | 0.204 |
| Uncinate fasciculus R | -0.400 ± 0.321 | -0.405 ± 0.300 | 0.149 | 0.881 | 0.941 | 0.017 |
| Uncinate fasciculus L | -0.298 ± 0.391 | -0.347 ± 0.34 | 1.184 | 0.237 | 0.620 | 0.131 |
| Tapetum R | 0.893 ± 1.162 | 0.935 ± 1.182 | -0.32 | 0.749 | 0.941 | 0.036 |
| Tapetum L | 0.690 ± 1.082 | 0.819 ± 1.119 | -1.039 | 0.300 | 0.720 | 0.118 |

The Z score of radial diffusivity for 48 white matter brain regions that represent the major fasciculi are presented as the mean ± standard deviation.

*ROI,* regions of interest; *p.adj*, *p* value adjusted by FDR method; *R*, right; *L*, left.

^a^ The *p value* was obtained by the two-sample *t* test

**Table S7. Comparison of the mean fractional anisotropy between the lipid-based subgroups**

| **ROI** | **Cluster 1** | **Cluster 2** | ***t*-statistic** | ***p value*** | ***p.adj*** | **Cohen's d** |
| --- | --- | --- | --- | --- | --- | --- |
| Middle cerebellar peduncle | -0.412 ± 0.393 | -0.427 ± 0.396 | 0.331 | 0.741 | 0.949 | 0.037 |
| Pontine crossing tract | -0.303 ± 0.552 | -0.384 ± 0.556 | 1.289 | 0.198 | 0.715 | 0.146 |
| Genu of corpus callosum | 0.49 ± 0.485 | 0.403 ± 0.504 | 1.551 | 0.122 | 0.586 | 0.176 |
| Body of corpus callosum | 0.971 ± 0.446 | 0.948 ± 0.415 | 0.470 | 0.639 | 0.944 | 0.053 |
| Splenium of corpus callosum | 1.976 ± 0.573 | 1.988 ± 0.441 | -0.207 | 0.836 | 0.965 | 0.023 |
| Fornix (column and body of the fornix) | -0.214 ± 0.885 | -0.439 ± 0.794 | 2.386 | 0.018^*^ | 0.586 | 0.266 |
| Corticospinal tract R | 0.041 ± 0.653 | 0.074 ± 0.606 | -0.457 | 0.648 | 0.944 | 0.051 |
| Corticospinal tract L | 0.089 ± 0.693 | 0.097 ± 0.698 | -0.102 | 0.919 | 0.965 | 0.011 |
| Medial lemniscus R | 0.553 ± 0.5 | 0.543 ± 0.608 | 0.159 | 0.874 | 0.965 | 0.018 |
| Medial lemniscus L | 0.568 ± 0.514 | 0.492 ± 0.551 | 1.260 | 0.209 | 0.715 | 0.143 |
| Inferior cerebellar peduncle R | -0.63 ± 0.556 | -0.604 ± 0.627 | -0.387 | 0.699 | 0.949 | 0.044 |
| Inferior cerebellar peduncle L | -0.631 ± 0.555 | -0.627 ± 0.613 | -0.069 | 0.945 | 0.965 | 0.008 |
| Superior cerebellar peduncle R | 0.173 ± 0.601 | 0.132 ± 0.478 | 0.684 | 0.495 | 0.913 | 0.075 |
| Superior cerebellar peduncle L | 0.378 ± 0.544 | 0.413 ± 0.497 | -0.594 | 0.553 | 0.944 | 0.066 |
| Cerebral peduncle R | 1.538 ± 0.301 | 1.522 ± 0.304 | 0.456 | 0.649 | 0.944 | 0.051 |
| Cerebral peduncle L | 1.764 ± 0.323 | 1.759 ± 0.336 | 0.138 | 0.891 | 0.965 | 0.016 |
| Anterior limb of internal capsule R | 0.361 ± 0.312 | 0.426 ± 0.333 | -1.788 | 0.075 | 0.586 | 0.203 |
| Anterior limb of internal capsule L | 0.461 ± 0.288 | 0.476 ± 0.282 | -0.482 | 0.630 | 0.944 | 0.054 |
| Posterior limb of internal capsule R | 1.419 ± 0.309 | 1.471 ± 0.286 | -1.558 | 0.120 | 0.586 | 0.174 |
| Posterior limb of internal capsule L | 1.928 ± 0.304 | 2.000 ± 0.348 | -1.918 | 0.056 | 0.586 | 0.220 |
| Retrolenticular part of internal capsule R | 0.811 ± 0.29 | 0.866 ± 0.323 | -1.591 | 0.113 | 0.586 | 0.182 |
| Retrolenticular part of internal capsule L | 0.998 ± 0.292 | 1.025 ± 0.269 | -0.855 | 0.393 | 0.858 | 0.095 |
| Anterior corona radiata R | -0.905 ± 0.278 | -0.895 ± 0.297 | -0.317 | 0.751 | 0.949 | 0.036 |
| Anterior corona radiata L | -0.935 ± 0.245 | -0.955 ± 0.262 | 0.699 | 0.485 | 0.913 | 0.079 |
| Superior corona radiata R | -0.437 ± 0.287 | -0.398 ± 0.325 | -1.125 | 0.261 | 0.781 | 0.129 |
| Superior corona radiata L | -0.236 ± 0.343 | -0.194 ± 0.347 | -1.085 | 0.279 | 0.781 | 0.123 |

**Table S7 (continued). Comparison of the mean fractional anisotropy between the lipid-based subgroups**

| **ROI** ^a^ | **Cluster 1** | **Cluster 2** | ***t*-statistic** | ***p value*** | ***p.adj*** | **Cohen's d** |
| --- | --- | --- | --- | --- | --- | --- |
| Posterior corona radiata R | -0.58 ± 0.31 | -0.568 ± 0.324 | -0.353 | 0.724 | 0.949 | 0.040 |
| Posterior corona radiata L | -0.929 ± 0.313 | -0.890 ± 0.343 | -1.057 | 0.291 | 0.781 | 0.121 |
| Posterior thalamic radiation R | 0.922 ± 0.387 | 0.828 ± 0.381 | 2.155 | 0.032^*^ | 0.586 | 0.243 |
| Posterior thalamic radiation L | 0.629 ± 0.358 | 0.612 ± 0.351 | 0.425 | 0.671 | 0.948 | 0.048 |
| Sagittal stratum R | 0.261 ± 0.343 | 0.303 ± 0.366 | -1.042 | 0.298 | 0.781 | 0.119 |
| Sagittal stratum L | 0.137 ± 0.348 | 0.109 ± 0.340 | 0.718 | 0.473 | 0.913 | 0.081 |
| External capsule R | -1.360 ± 0.331 | -1.396 ± 0.300 | 1.019 | 0.309 | 0.781 | 0.114 |
| External capsule L | -1.209 ± 0.3 | -1.241 ± 0.271 | 0.982 | 0.327 | 0.785 | 0.109 |
| Cingulum (cingulate gyrus) R | -1.103 ± 0.534 | -1.066 ± 0.502 | -0.625 | 0.532 | 0.944 | 0.070 |
| Cingulum (cingulate gyrus) L | -1.061 ± 0.535 | -1.055 ± 0.531 | -0.092 | 0.927 | 0.965 | 0.010 |
| Cingulum (hippocampus) R | -1.404 ± 0.366 | -1.333 ± 0.38 | -1.663 | 0.097 | 0.586 | 0.189 |
| Cingulum (hippocampus) L | -1.379 ± 0.463 | -1.278 ± 0.424 | -2.024 | 0.044^*^ | 0.586 | 0.226 |
| Fornix (cres)/Stria terminalis R | -0.582 ± 0.49 | -0.569 ± 0.477 | -0.252 | 0.801 | 0.965 | 0.028 |
| Fornix (cres)/Stria terminalis L | -0.083 ± 0.426 | -0.094 ± 0.408 | 0.218 | 0.828 | 0.965 | 0.024 |
| Superior longitudinal fasciculus R | -0.06 ± 0.316 | -0.012 ± 0.295 | -1.408 | 0.160 | 0.698 | 0.158 |
| Superior longitudinal fasciculus L | -0.214 ± 0.299 | -0.215 ± 0.319 | 0.025 | 0.980 | 0.98 | 0.003 |
| Superior fronto-occipital fasciculus R | -1.062 ± 0.494 | -1.033 ± 0.573 | -0.481 | 0.631 | 0.944 | 0.055 |
| Superior fronto-occipital fasciculus L | -1.057 ± 0.577 | -1.156 ± 0.488 | 1.668 | 0.096 | 0.586 | 0.184 |
| Uncinate fasciculus R | -0.111 ± 0.532 | -0.061 ± 0.596 | -0.778 | 0.437 | 0.913 | 0.089 |
| Uncinate fasciculus L | -0.183 ± 0.587 | -0.125 ± 0.585 | -0.888 | 0.375 | 0.858 | 0.100 |
| Tapetum R | 0.368 ± 0.521 | 0.284 ± 0.600 | 1.309 | 0.192 | 0.715 | 0.150 |
| Tapetum L | 0.248 ± 0.53 | 0.243 ± 0.601 | 0.076 | 0.940 | 0.965 | 0.009 |

The Z score of fractional anisotropy for 48 white matter brain regions that represent the major fasciculi are presented as the mean ± standard deviation.

*ROI,* regions of interest; *p.adj*, *p* value adjusted by FDR method; *R*, right; *L*, left.

^a^ The *p value* was obtained by the two-sample *t* test

**Table S8. Association of identified lipids and brain white matter alterations in psychiatric patients.**

| Lipids | FA | | | RD |
| --- | --- | --- | --- | --- |
|  | Fornix | Posterior thalamic radiation R | Hippocampus L | Genu of corpus callosum |
| DGTS 16:0/18:1 | -0.11 | -0.11 | 0.12^*^ | 0.18^**^ |
| 4-Amino-3-methylbutanoic acid | -0.06 | 0.00 | 0.01 | 0.04 |
| 9,12-Octadecadienal | -0.05 | -0.05 | 0.12^*^ | 0.08 |
| 20-oxo-22,23,24,25,26,27-hexanorvitamin D3 | 0.00 | 0.07 | -0.03 | 0.00 |
| Hexadecandioic acid | 0.05 | 0.02 | 0.11^*^ | 0.11^*^ |
| 12-Tridecynoic acid | 0.04 | -0.01 | 0.11 | 0.11 |
| OxPC 16:0-18:1+2O | 0.01 | 0.05 | -0.03 | -0.08 |
| Cyclopentaneoctanoic acid | -0.08 | -0.10 | 0.00 | 0.08 |
| Caprylic acid | -0.12^*^ | -0.08 | 0.02 | 0.09 |
| 10-Nitro-9Z,12Z-octadecadienoic acid | -0.03 | -0.03 | -0.02 | 0.02 |

The correlation coefficient are presented.

*R*, right; *L*, left.

^*^*p* < 0.05; ^**^*p* < 0.01;
